# Supplementary material for: Observation of gapped Dirac cones in a two-dimensional Su-Schrieffer-Heeger lattice
Source: Nat Commun. 2022 Nov 16;13:7000. doi: 10.1038/s41467-022-34043-9 (PMC9668907; doi:10.1038/s41467-022-34043-9)
Supplement: Supplementary file 1 — Supplementary Information [file 41467_2022_34043_MOESM1_ESM.docx]

**Supplementary Information**

**Observation of Gapped Dirac Cones in a Two-Dimensional** **Su-Schrieffer-Heeger Lattice**

Daiyu Geng,^1,2,#^ Hui Zhou,^1,2,#^ Shaosheng Yue,^1,2^ Zhenyu Sun,^1,2^ Peng Cheng,^1,2^ Lan Chen,^1,2,3^ Sheng Meng,^1,2,3,4*^ Kehui Wu,^1,2,3,4*^ Baojie Feng,^1,2,4*^

*^1^Institute of Physics, Chinese Academy of Sciences, Beijing, 100190, China*

^2^*School of Physical Sciences, University of Chinese Academy of Sciences, Beijing, 100049, China*

^3^*Songshan Lake Materials Laboratory, Dongguan, Guangdong, 523808, China*

*^4^Interdisciplinary Institute of Light-Element Quantum Materials and Research Center for Light-Element Advanced Materials, Peking University, Beijing, 100871, China*

^#^These authors contributed equally to this work.

^*^Corresponding author. E-mail: smeng@iphy.ac.cn; khwu@iphy.ac.cn; bjfeng@iphy.ac.cn.


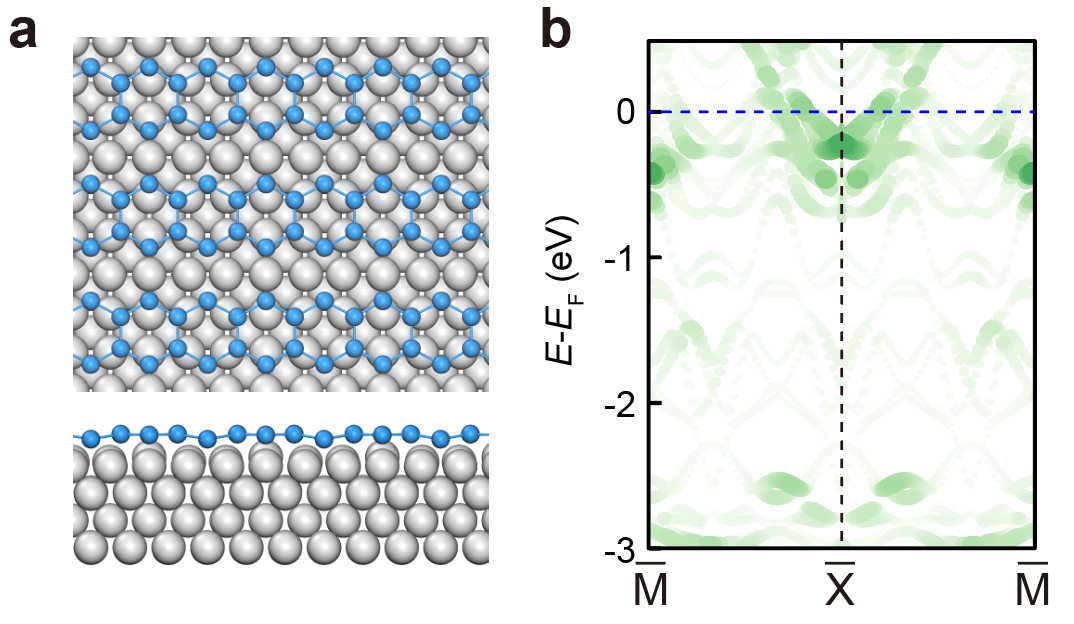


**Supplementary Fig. 1: The hexagonal structure proposed in Ref. [31] of the main text.** (a) Relaxed structure model of Si/Ag(001). (b) DFT calculated band structure of Si/Ag(001) along the $\bar{M}-\bar{X}-\bar{M}$ direction of Ag(001), which contradict with our ARPES measurement results.


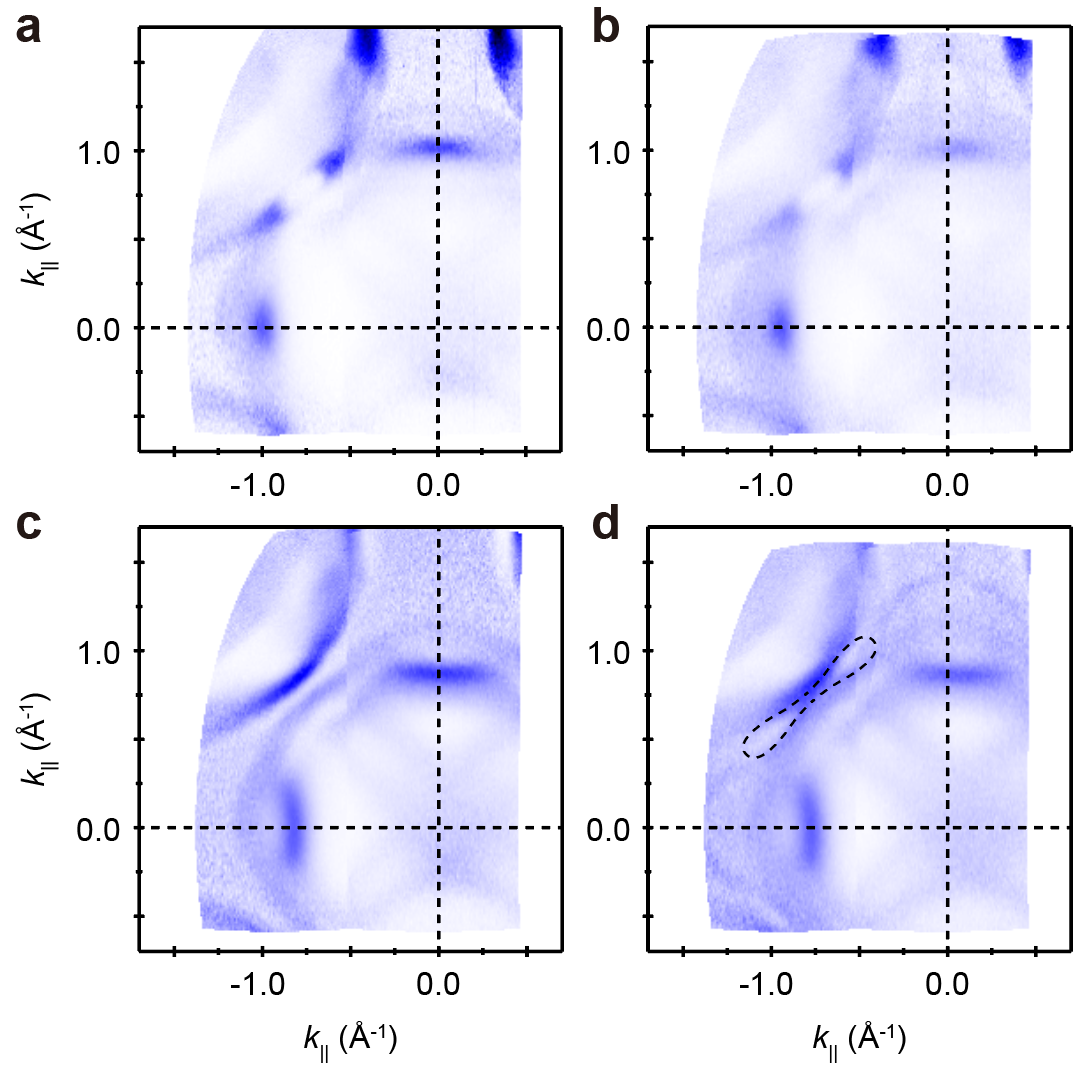


**Supplementary Fig. 2: ARPES measurements of constant energy contours.** (a,b) ARPES intensity plots of pristine Ag(001) and Si/Ag(001) at the Fermi level, respectively. (c,d) The same as (a,b) but for *E*_B_~1.1 eV. Dash lines in (d) serve as a guide to the eye.


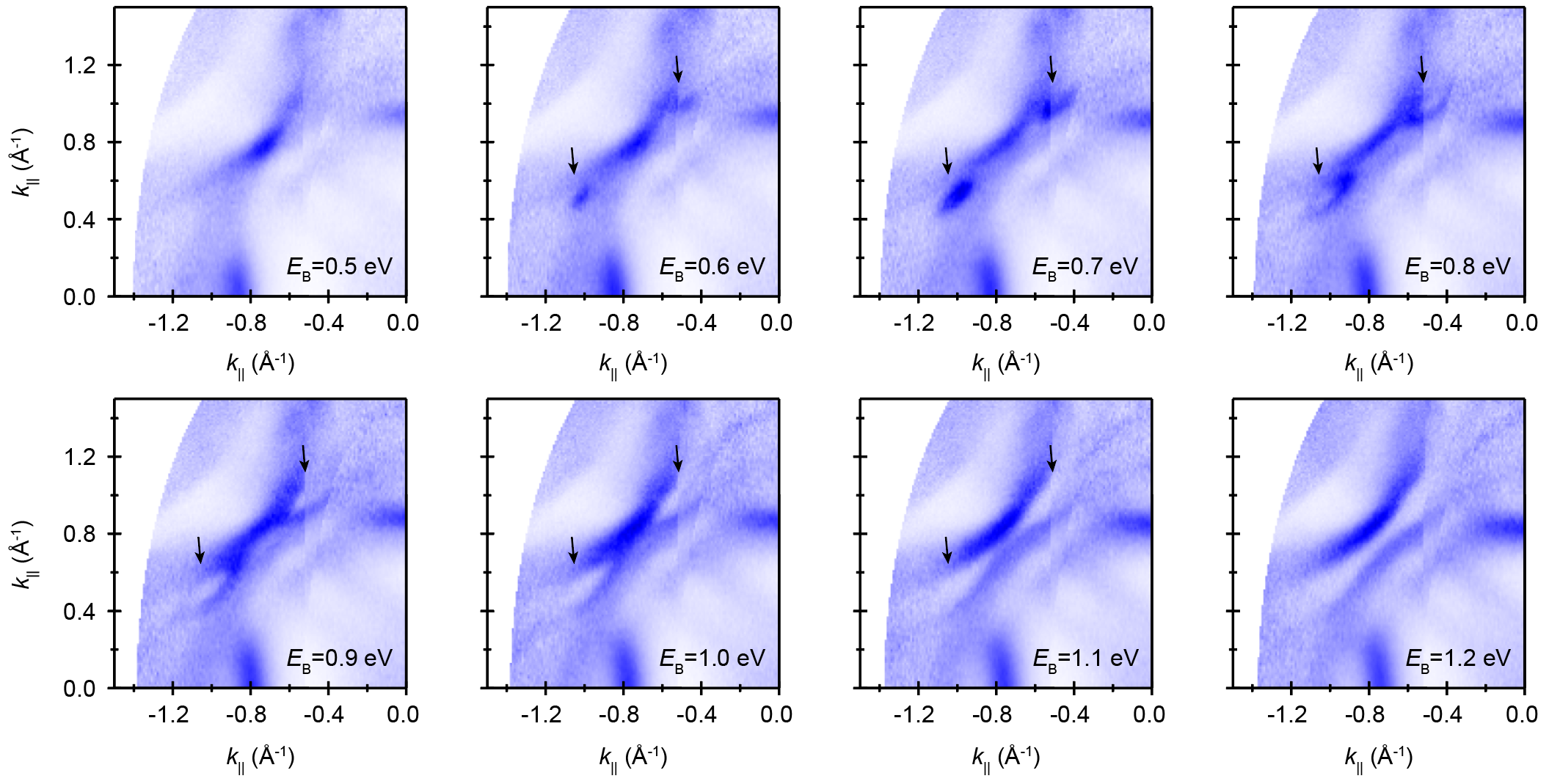


**Supplementary Fig. 3:** **Energy** **evolution of constant energy contours.** The binding energies are from *E*_B_=0.5 eV to *E*_B_=1.2 eV. Dot-like features emerge from 0.6 eV and become pockets at higher binding energies. Finally, neighboring pockets merge at approximately 1.0 eV. Black arrows indicate the evolution of the Dirac cones.


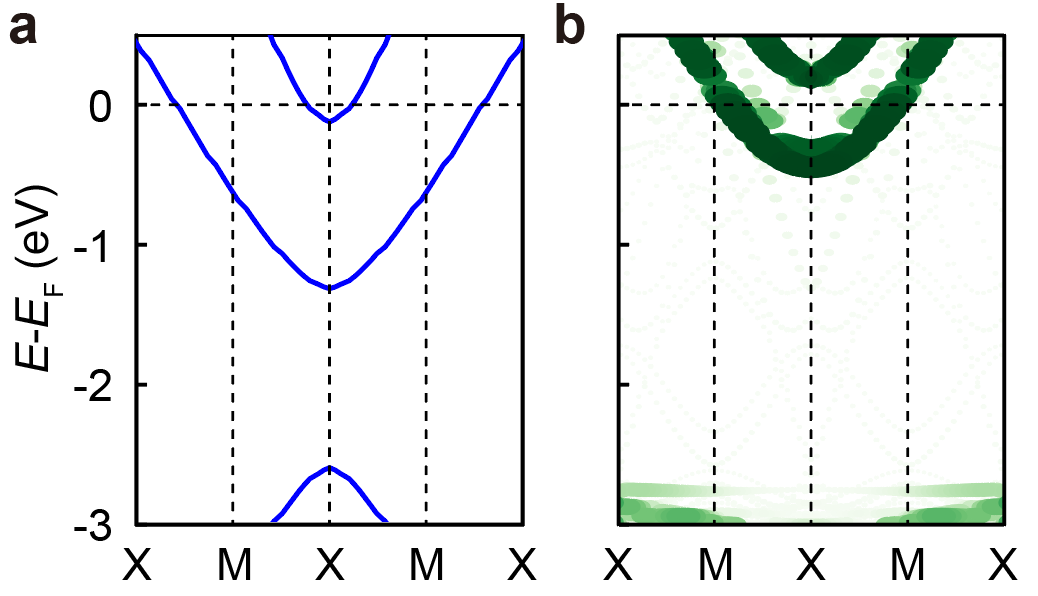


**Supplementary Fig. 4:** **DFT calculated band structures of pristine and reconstructed Ag(001) based on a 3×3 supercell.** (a) Calculated band structures of pristine Ag(001) along the $\bar{M}-\bar{X}-\bar{M}$ direction. The momentum range was adjusted to be consistent with Cut 2 of Fig. 3 in the main text. (b) Unfolded band structures of 3×3-reconstructed Ag(001) without Si. The effective band structure was unfolded to the first BZ of Ag(001). Four layers of Ag atoms were considered in the calculations.


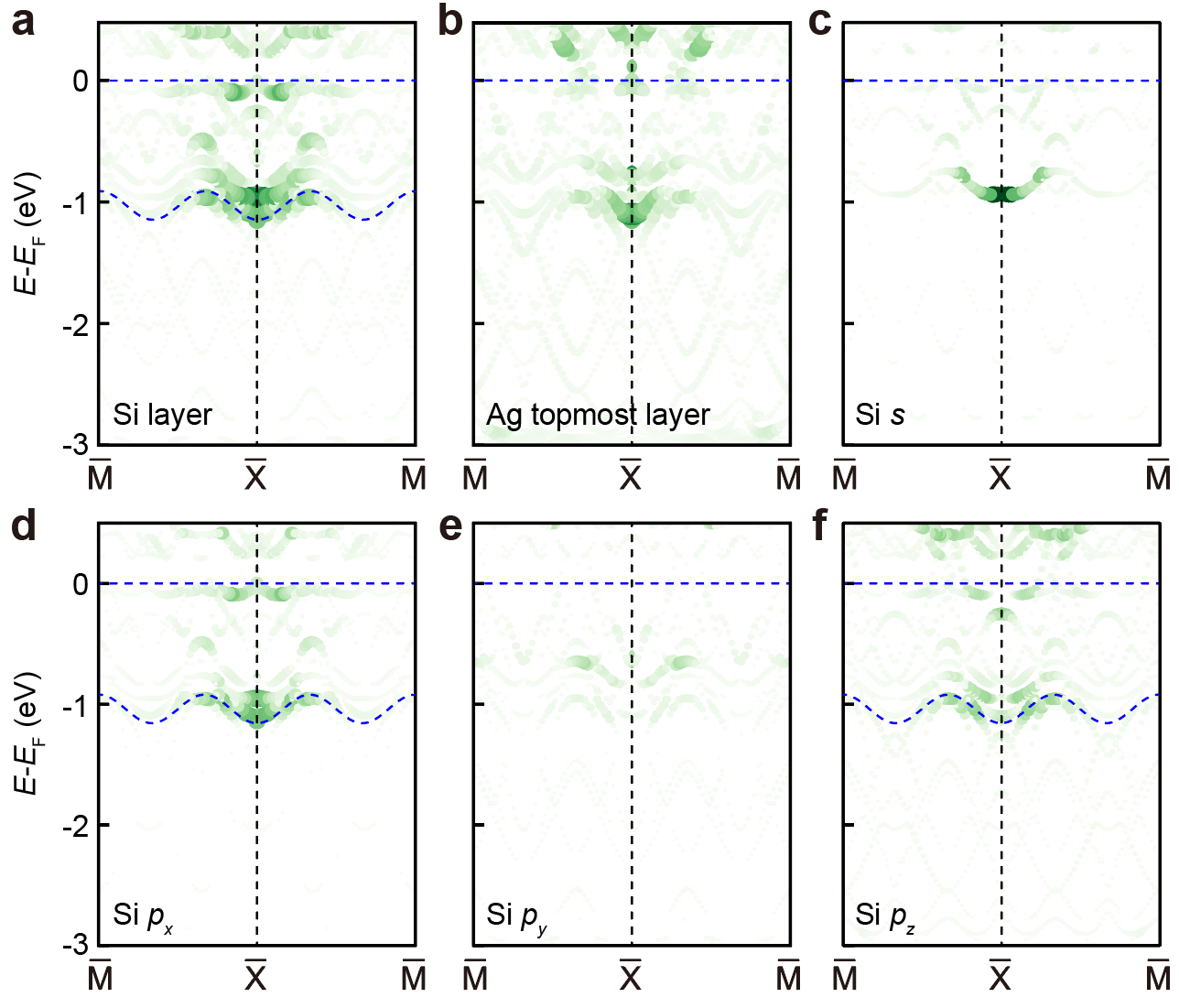


**Supplementary Fig. 5: Orbital projection of unfolded band structures.** (a,b) DFT calculated band structures projected to the Si and topmost Ag layer, respectively. The calculation is based on the structure model shown in Fig. 2(c) of the main text. (c-f) Calculated band structures projected to the Si *s*, *p_x_*, *p_y_*, and *p_z_* orbitals, respectively, along the $\bar{M}-\bar{X}-\bar{M}$ direction of Ag(001).


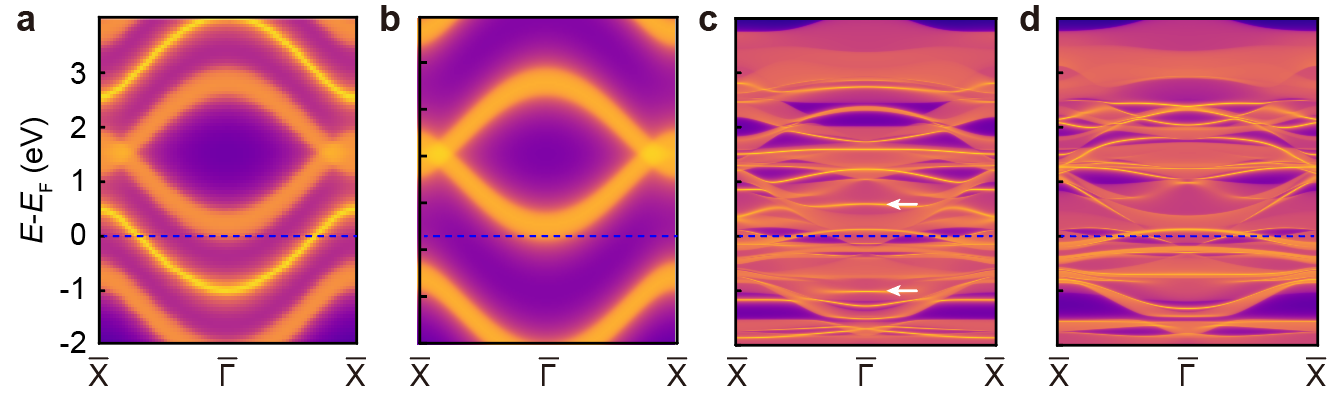


**Supplementary Fig. 6:** **Calculation of edge states.** (a,c) TB and DFT calculated edge spectrum of the nontrivial edge obtained by cutting the system through the centers of the blue bonds in the *y* direction (the Wannier center). (b,d) TB and DFT calculated edge spectrum of the trivial edge obtained by cutting the system through the centers of the green bonds in the *y* direction. White arrows in (c) indicate topological edge states. The substrate was included in DFT calculations.


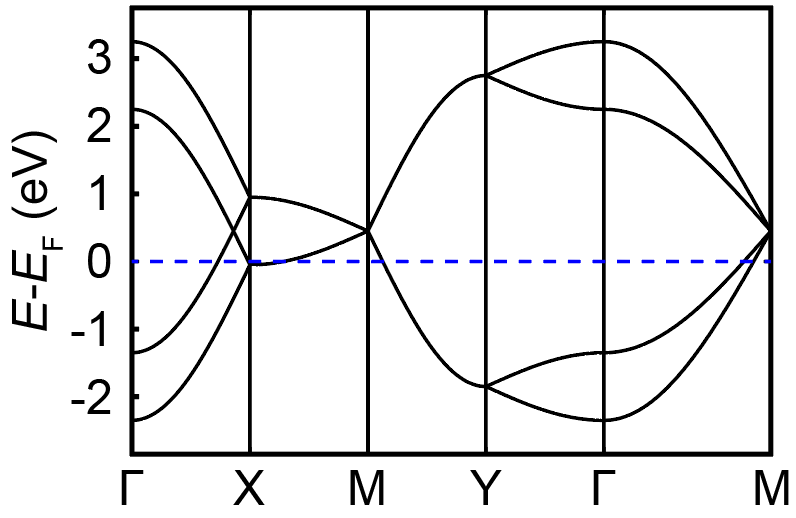


**Supplementary Fig. 7:** **Creation of gapless and anisotropic Dirac cones.** TB calculated band structure with parameters: $t_{1\pi}=t_{3\pi}=0.25$ eV, $t_{2\sigma}=t_{2\pi}=1.15$ eV, and $U_{1}=U_{2}=0.45$ eV. A gapless and anisotropic Dirac cone appears at the M point.
